# Arrears of Contributions and the Compulsory System

**Published:** 1915-08-14

**Authors:** 


### NATIONAL INSURANCE ACT DEVELOPMENTS.

### Arrears of Contributions and the Compulsory System.

Of all troublesome matters—and they are many—in the practical administration of national health insurance those connected with arrears of contribution are the most troublesome. The worst of it is, too, that instead of matters becoming more simple with the lapse of time they are now more complicated than ever. The subject is at present causing an immense amount of anxiety and annoyance to officials of approved societies, and they are agitating, with good reason, for a simplification of the regulations which it is their duty to carry out. The regulations are far too technical for us to attempt to give their bare outline. It is sufficient, however to say that there are no fewer than sixty-five numbered sections in the circular (37 M/A.G.D.) which has recently been issued giving instructions as to the methods of dealing with the arrears for the purposes of record in the insurance books of members, and in the accounts of the societies. Furthermore, we learn that in order to make these instructions intelligible to its district representatives one large approved society has had to print a pamphlet containing something like twelve thousand words.

Is it surprising in these circumstances that the secretaries of small local approved societies, many of whom are only able to give their spare time in the evenings to the work, cannot grasp all the complicated directions contained in the official circular? The natural consequence is that they make mistakes in accounting, which, it may be, are not discovered by the Insurance Commissioners, or by the auditors appointed by them, until long after the dates for completing the accounts, and the task of rectification is not rendered the easier by being deferred.

### The Effect on the Member.

The effect on the insured member, with whose interests we are mainly concerned, is indirect, but hardly less important for that reason than the effect on the officials of the approved societies. The ordinary member, it is true, does not have to read pages of instructions, but he may suffer loss through the omission on the part of the secretary of his society to point out the necessary procedure appropriate to his case. Moreover, with harassed officials the whole body of members must necessarily suffer, through lack of attention that should be devoted to their interests, while financially it is open to question whether the saving on the benefit funds due to a rigid observance of the regulations is not more than counterbalanced by the loss of profit which might otherwise be obtained from the management fund.

Matters are apparently too far advanced to hope for any relief during the current year, but it is not too early to devise a plan with the object of simplifying the regulations at the earliest possible moment. We therefore put forward the following suggestions in the hope that they may be of some assistance in securing the fullest possible advantages to insured persons from the national insurance scheme.

### How the Difficulties Arise.

The difficulties appear to arise from the fact that in spite of the compulsory character of the scheme and the payment of contributions by employers, it was thought to be necessary when the Act of 1911 was framed to take account of arrears of contributions by reducing the rates of benefit corresponding in some degree to the extent of the arrears. The framers of the Act were, doubtless, guided by the fact that friendly societies whose membership was voluntary, invariably had rules governing the right to benefit when contributions were not paid to date. They departed, however, from the conditions usually imposed in several important particulars. The conditions were, generally, that the arrears must be paid before benefit could be drawn, or that the arrears should be deducted from the benefits payable, and that if the arrears should exceed a prescribed limit the right to benefit would lapse altogether.

The plan originally adopted in the national insurance scheme, it will be remembered, was that if the average arrears, taking into account the duration of insurance, exceeded certain limits, the ordinary rates of benefit were to be reduced on a sliding scale, corresponding to the average arrears. This plan, in the opinion of the Insurance Commissioners, precluded the issue of half-yearly contribution cards, and, consequently, as the demand for half-yearly cards, instead of quarterly cards, could not be resisted, a revised method of dealing with the arrears had to be evolved.

The revised plan, under the Act of 1913, is, briefly, that the penalty of arrears in one contribution year is a reduction of the rate of sickness benefit to which the member is entitled, should he be ill, in the succeeding benefit year. At first it was thought that the revised plan was an improvement upon the original. Owing, however, to the fact that three contributions are allowed each year to be unpaid without any penalty resulting, and that these contributions, if paid, are carried forward as reserve contributions, while the insured person is allowed to pay arrears within a certain specified period, it is easy to see that the revised plan entails, at its best, quite a considerable amount of skilled labour correctly to mark the insurance books of members, and to keep the records at the offices of the approved societies. It is to be particularly noted in this connection that under the revised scheme every insurance book must be marked each year, whether the member claims benefit or not, and the state of each account in the matter of arrears must receive individual attention in the records of the society.

When, as at the present time, there are numerous complications in the rates of contributions, due to changes in status from one class of insurance to another, the plan becomes so overweighted with detail that it is apparently on the verge of breaking down altogether.

A Suggested Simplification.

In this elaboration of detail it appears that the essential difference between a voluntary system of insurance and a compulsory system has escaped attention.

In a voluntary system there must of necessity be a penalty for arrears, but when the system is compulsory, and the contributions are paid by employers, it does seem, upon closer consideration, that there is not the same necessity, and certainly that if any penalty at all is to be imposed the scheme could, without injustice to the vast majority of the insured, be made very much more simple than the one at present in force.

The vast majority of insured persons are regularly employed, and consequently no question of arrears has to be considered so far as they are concerned. There is, however, a small minority whose employment is irregular, or who are only occasionally employed within the meaning of the Act. At the present time these persons are entitled to pay as arrears their own contributions (4d. men, and 3d. women, with minor variations) instead of the full contribution, including the employer's share, which would have been payable if they had been at work.

It would surely be possible to enact that every approved society should have the right to expel any member whose contributions fall short of the full number payable in any given year by, say, eight or twelve weeks, or such other number of weeks that might be desirable, after giving such member due warning, and a limited period, say three months, in which the arrears might be paid. If this were done, no other direct penalty for arrears would be necessary.

When the expulsion became operative the member would either have to gain admission to another society—which might be difficult in the circumstances—or would be forced to become a Deposit Contributor. No real hardship would be caused by this action, for such an allowance as eight or twelve weeks for a single year should be ample to secure that those who are *bonâ fide* employed do not lose benefit; while have those who are only intermittently employed a genuine claim to the benefits of the compulsory insurance, when they would as Deposit Contributors receive the benefit of the contributions that are actually paid?

The Financial Effect of the Scheme.

It might be urged that the finances of approved societies would be jeopardised by such an alteration, but it is hardly feasible to suppose that any one society, or class of societies, would be adversely affected to any considerably greater extent than any other society, or class of society. Moreover, the fact that arrears within the limit stipulated would tend adversely to affect the funds of the societies would lead to greater efficiency in the supervision of contributions, and the societies would, as far as possible, see that arrears were not allowed to accumulate. Furthermore, although there might be a small actual loss in contributions, on account of the arrears, this should to a large extent, if not in total, be counterbalanced by the saving in the cost of administration, owing to the simplification of the system. There is above all the corrective for any expenditure beyond the real financial ability of the society to be found in the provision for periodical valuations. If a society had, through lack of supervision, allowed its members persistently to fall into arrear, the members would suffer by reason of the deficiency that would be shown on valuation.

We commend the suggestions herein contained to the notice of all who are interested in the practical administration of the National Insurance Act. They are suggestions which would apparently entail an amendment of the law, if they are to be carried into effect, but if the powers of the Insurance Commissioners are not at present wide enough to ensure practical administrative efficiency, those powers must be enlarged until such efficiency is obtainable.

